# Nursing Care Coordination for Patients with Complex Needs in Primary Healthcare: A Scoping Review

**DOI:** 10.5334/ijic.5518

**Published:** 2021-03-19

**Authors:** Marlène Karam, Maud-Christine Chouinard, Marie-Eve Poitras, Yves Couturier, Isabelle Vedel, Nevena Grgurevic, Catherine Hudon

**Affiliations:** 1 Department of Family Medicine and Emergency Medicine, Université de Sherbrooke, Quebec, Canada; 2 Université de Montréal, Faculty of Nursing, Quebec, Canada; 3 Department of Social Work, Université de Sherbrooke, Quebec, Canada; 4 Centre de recherche du CHUS, Quebec, Canada; 5 Department of Family Medicine, McGill University, Quebec, Canada

**Keywords:** care coordination, integrated care, primary healthcare, complex health and social care needs, registered nurses, review

## Abstract

**Introduction:**

Millions of people worldwide have complex health and social care needs. Care coordination for these patients is a core dimension of integrated care and a key responsibility for primary healthcare. Registered nurses play a substantial role in care coordination. This review draws on previous theoretical work and provides a synthesis of care coordination interventions as operationalized by nurses for complex patient populations in primary healthcare.

**Methodology:**

We followed Arksey and O’Malley’s methodological framework for scoping reviews. We carried out a systematic search across CINAHL, MEDLINE, Scopus and ProQuest. Only empirical studies were included. We performed a thematic analysis using deductive (the American Nurses Association Framework) and inductive approaches. Findings were discussed with a group of experts.

**Results:**

Thirty-four articles were included in the synthesis. Overall, nursing care coordination activities were synthesized into three categories: those targeting the patient, family and caregivers; those targeting health and social care teams; and those bringing together patients and professionals. Interpersonal communication and information transfer emerged as cross-cutting activities that support every other activity. Our results also brought to light the nurses’ contribution to care coordination efforts for patients with complex needs as well as critical components that should be present in every care coordination intervention for this clientele. These include an increased intensity and frequency of activities, relational continuity of care, and home visits.

**Conclusion:**

With the growing complexity of patient’s needs, efforts must be directed towards enabling the primary healthcare level to effectively play its substantial role in care coordination. This includes finding primary care employment models that would facilitate multidisciplinary teamwork and the delivery of integrated care, and guarantee the delivery of intensive yet efficient coordinated care.

## Introduction

Over the last two decades, integration has become a major concern for many governments and healthcare systems [1,2,3,4]. With limited financial resources, aging populations, and comorbid chronic diseases [5,6,7], many countries have recognized the need to move from fragmented and discontinued care towards a more integrated healthcare system [8]. Studies showed the potential of integrated care to improve continuity of care, accessibility, quality and safety of care, as well as cost effectiveness of services [9]. Care coordination around patients’ needs has been acknowledged as a core dimension of integration that facilitates the provision of comprehensive and seamless care [10]. It has also been recognized as a key responsibility for primary healthcare [11].

As complexity grows, so does the need for a stronger primary healthcare able to deliver more care in the community and coordinate care within primary care and across care levels [12,13]. Millions of people worldwide have complex needs that span beyond what the healthcare system typically provides [14]. Fragmentation of health and social care services causes patients with complex needs to bear the major responsibility for navigating their own pathway through services and providers [15] and they experience systems as being confusing and overwhelming [16]. For these patients, care coordination and integration of health and social care services are even more relevant.

Although the aim of care coordination is widely agreed upon, there is still a lack of global consensus around a single conceptual model and much ambiguity in the definitions of care coordination [17]. The Agency for Healthcare Research and Quality defines care coordination as “*the deliberate organization of patient care activities between two or more participants (including the patient) involved in a patient’s care to facilitate the appropriate delivery of health care services”* [18]. A variety of approaches has been adopted to deliver coordinated care in practice. Case management is perhaps the most intensive intervention for caring for people with complex health and social needs [19]. Case management has been established as a targeted, community-based and proactive approach to care that involves case finding, assessment, care planning, and care coordination [19]. Patient navigation is another approach that has emerged within primary care as a means to link patients and families to primary care services, specialist care, and community-based health and social services to provide holistic patient-centred care [20]. The Agency for Healthcare Research and Quality has identified several other approaches and terms that are often used synonymously or in conjunction with care coordination, namely collaborative care, disease management, care management, and the Chronic Care Model [18]. However, irrespective of the approach that is adopted, an essential dimension of effective care coordination is the involvement of a multidisciplinary primary care team that functions as a cooperative, cohesive unit to provide the right care in the right place at the right time [16].

Within primary healthcare, the role of care coordinator can be undertaken by professionals who come from various backgrounds, including nursing, social work, physiotherapy and occupational therapy, as long as they are equipped with and trained on the necessary skills [19]. The choice of a designated care coordinator is often dependent on contextual factors, the population of interest, and the goals of the program. Indeed, a foundational element of care coordination is a holistic care perspective that includes addressing clinical/medical as well as the broader determinants of health [21]. It is this very perspective that gave both nurses and social workers their legitimate position in organizing and managing care for a complex population. However, one might be better equipped than the other depending on the patient’s condition and the disciplinary expertise this condition primarily calls for. For instance, social care expertise is particularly important for patients in the rehabilitation and reablement phases and those with a functional decline [19], while nursing clinical expertise may be more relevant for patients suffering from serious pathologies such as cancer [22]. In either case, health and social care professionals still have to work collaboratively and use their unique skills and disciplinary expertise as needed. Primary care practices that have the capacity (e.g. structure, resources) have implemented a team-based model of care coordination where the social worker and registered nurse carry out a joint patient assessment. This care coordination model proved effective in increasing communication between health and social services and in improving care for complex patients such as older health consumers [23].

This scoping review focuses on the role of registered nurses in care coordination. Nurse-led care coordination interventions proved effective in improving access to appropriate treatment [24]; reducing costs [25,26,27]; improving clinical outcomes [27,28] and quality of care [29]; improving communication between staff [30]; increasing safety of vulnerable patients during transition [29]; and reducing their unplanned readmissions [19,31]. It should be noted that, depending on local needs and resources, nurses might be employed to undertake exclusive care coordination activities [32,33] or they might combine care coordination with wider team management responsibilities or with clinical care provision [34].

Despite the substantial involvement of nurses in care coordination efforts, their contribution still has to be clearly “*defined, measured and reported to ensure appropriate financial and systemic incentives for the professional care coordination role*” [35].

To date, there persists a lack of knowledge about interventions and activities performed by registered nurses for patients with complex health and social care needs in primary healthcare. An in-depth analysis of how the nursing care coordination role for patients with complex needs is operationalized is clearly needed. The Agency for Healthcare Research and Quality also stressed the need for care coordination frameworks to be enriched by empirical data. In 2010, the Agency developed a framework and specified that “*this framework provides a starting place for understanding care coordination… and is intended to grow with the field… since care coordination is a rapidly growing evidence base field”* [36].

### Aim and research questions

This scoping review draws on previous theoretical work and aims to provide a synthesis of care coordination interventions as operationalized by nurses for patients with complex health and social care needs in primary healthcare. Our research questions are: What care coordination interventions are currently performed by registered nurses in primary healthcare? Who are the target complex patient populations? What activities do these interventions involve?

### Conceptual Framework

In 2013, the American Nurses Association developed a framework for measuring nurses’ contributions to care coordination [37]. This framework was based on several other theoretical frameworks including the one developed by the Agency for Healthcare Research and Quality for healthcare. The American Nurses Association’s framework has the strength of providing a roadmap for how conceptualization of nursing’s role in care coordination can be operationalized, quantified and measured, and includes thirteen constructs that comprise the nursing care coordination processes. Thus, we considered it a particularly suitable starting point for our review. However, care coordination activities described in the American Nurses Association Framework were cross- cutting, relevant to all settings of care and services, while we were specifically interested in the primary healthcare level and target complex patient populations.

## Methods

We followed the methodology proposed by Arksey and O’Malley [38] and further developed by Levac et al. [39]. This methodological framework offers six stages for carrying out scoping reviews: Identifying the research question; Identifying relevant studies; Selecting studies; Charting the data; Collating, summarizing, and reporting the results; Consulting experts.

**Figure 1:**
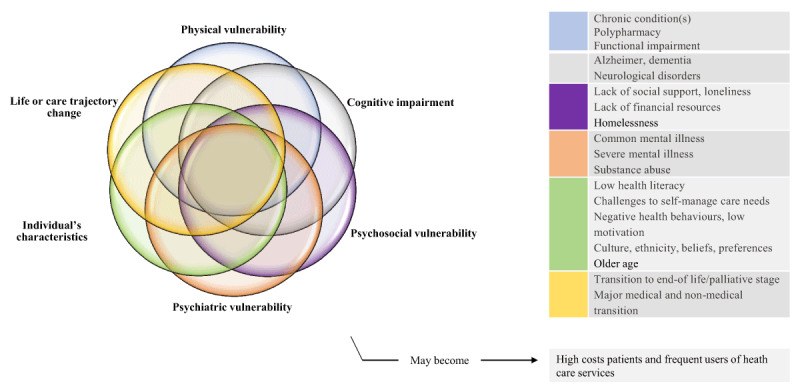
Complex health and social care needs. *Source*: Authors’ own elaboration. Key articles that guided this illustration include Garcia ME et al. 2018 [40]; Grembowski D, et al. 2014 [42]; Kuluski K, et al. 2017 [14].

1. **Identifying the research questions** (that we mentioned above).
2. **Identifying relevant studies:** We performed a literature search using the many terms that were identified by the Agency for Healthcare Research and Quality to describe care coordination interventions: “care coordination; case management; disease management; care management; care navigation; patient navigation; patient-centred medical home; and integrated care”. We combined these terms with “primary healthcare” or “primary care” and “nurses”. The search strategy, developed in consultation with an experienced medical librarian, may be found in Appendix 1. We conducted our search across the following databases: CINAHL, MEDLINE, Scopus, and ProQuest (dissertations and theses), for articles published over the past 15 years (between 2004 and 2019).
3. **Study selection:** In order to be included, articles had to report on an empirical study describing a nurse-led care coordination intervention in primary health care for adult patients with complex needs. Articles had to be written in English or French. Articles that described an advanced nursing practice, or a specialized (hospital, emergency department) level of care-based intervention were excluded. Articles were also excluded if the nurses activities and their distinct role within the multidisciplinary team were poorly described. No restrictions were set in relation to the nurses’ employment arrangement. Two independent reviewers (MK and NG) assessed the selected abstracts for inclusion. Disagreements were resolved by a consensus-based discussion between both investigators, or by a third reviewer (CH). For the purpose of this selection, we had to clarify what we mean by “patients with complex health and social care needs”. In the light of an extensive review of the existing literature on the subject, we put together a series of six areas of “vulnerability”, the combination of which define complex needs (***Figure 1***). Consequently, we adapted Kuluski’s definition of complex needs [14] as follows: “*Complex health and social care needs result from multiple concurrent chronic conditions, functional and cognitive impairments, mental health challenges and social vulnerability, the individual’s characteristics, or a major change in his life or care trajectory*”. Patients presenting either a combination of two or more elements, or a major vulnerability in one of these six areas (i.e. severe mental illness; transition to palliative stage) were considered as having complex needs. Very often, this combination of multiple elements (i.e. chronic conditions and social, mental health, and economic stressors) contributes to increased care utilization [40], therefore high-cost patients and frequent users were also included in our synthesis.
4. **Charting the data:** Two authors (MK and NG) extracted the following variables from each selected article: the study design, objectives, care coordination intervention, target population, and context. Care coordination activities were examined and extracted according to the American Nurses Association Framework for measuring nurses’ contributions to care coordination [37].
5. **Collating, summarizing and reporting the results:** We performed a thematic analysis using both deductive (using the American Nurses Association Framework) and inductive approaches. We used an initial list of predefined codes but also identified emergent codes through repeated examination of each care coordination intervention. We then identified patterns and relationships in order to organize codes into interrelated categories, then summarized the data [41]. Codes and categories were constantly discussed between investigators and evolved throughout the analysis phase. NVivo 12 software was used for data organization and management.
6. **Consultation:** Preliminary findings were presented and discussed on two separate occasions with senior and junior researchers in the field of integrated care during a face-to-face scientific meeting and a live videoconference. The latter also included a patient-research-partner. Researchers had a range of expertise (in nursing science, general medicine, mental health, social work and anthropology) and helped to refine the results and validate the implications for future research and practice.

## Results

**Figure 2:**
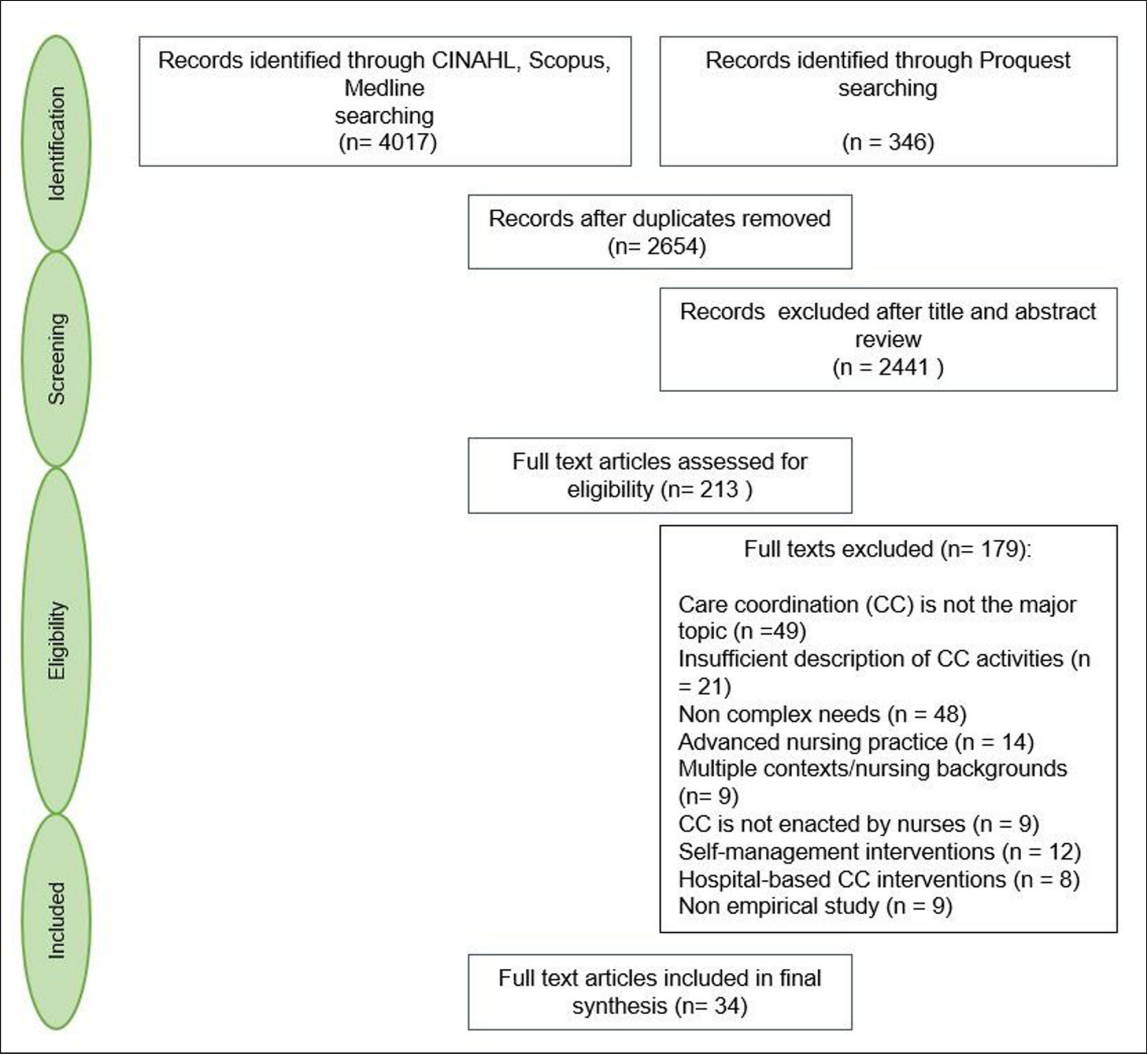
Flow diagram detailing search results and study selection.

Two hundred and thirteen full-text articles were screened; 34 articles met the inclusion criteria and were included in the synthesis. Our search results and the retrieval and selection of studies are presented in the flow diagram below (***Figure 2***). The 34 studies included in our synthesis reported on 26 different interventions. When the same care coordination intervention was reported in several articles, only the paper that provided a full description of this intervention was coded. However, the content of these other papers contributed to the consolidation and further clarification of the extracted data. ***Table 1*** presents all included papers, their main features and results.

**Table 1:** Included studies that address nursing care coordination for patients with complex needs in primary healthcare.


AUTHORS, YEAR OF PUBLICATION	STUDY DESIGN/OBJECTIVES	CARE COORDINATION INTERVENTIONS	TARGET POPULATION	NURSE’S EMPLOYMENT ARRANGEMENT, LOCALISATION, CONTEXT	SUMMARY OF CARE COORDINATION ACTIVITIES

Aragonès & al; 2012	Cluster RCT/Assess the effectiveness of a multi-component programme to improve the management of depression	Case management	Patients with moderate to severe depression	Exclusive case management role; Primary care centres, Catalonia, Spain	Provide self-management, psychological and emotional support; encourage and monitor adherence to treatment; monitor side effects and clinical results; improve social support; coordinate care between patient, primary care and specialized care providers; document information and transfer to GP

Berra & al; 2007	Clinical trial and program dissemination project/Reflect on lessons learned in developing the “Heart to Heart” program	Case management	Low-income, ethnically-diverse populations at elevated risk of cardiovascular disease events	Exclusive case management role; Primary care clinics, California, USA	Provide intensive and individualized care and guideline-based pharmacotherapy; provide education and counseling; support behavior change; coordinate access to community resources; link and partner with GP and specialty services

Bleijenberg & al; 2016. (intervention described in Bleijenberg 2013)	Cluster RCT/Determine the effectiveness of a primary care program on the daily functioning of older people	Nurse-led care intervention	Community-dwelling frail people aged 60 and older	Exclusive care coordination role; General practices, the Netherlands	Assess patient’s needs at home; develop care plans; monitor and follow up; support caregivers; promote self management; coordinate care with GP and health care teams

Boyd & al; 2007 (same intervention as in Boyd 2008; Boult 2008; Boult 2011; and Boult 2013)	Pilot test and preliminary results of an RCT/Test the feasibility of a new model of health care designed to improve the quality of life and the efficiency of resource use	Guided care	Older patients with multimorbidity and functional disability and who had generated high insurance expenditures for health care	Exclusive care coordination role; Non-academic urban primary care practice, Baltimore, USA	Assess the global patient’s status at home; develop a plan of care; monitor, follow up and respond to change; communicate and coordinate the efforts of all involved health care professionals; support patient and caregivers’ activation, engagement and self-management using motivational interviewing; facilitate access to community resources; facilitate care transition

Coburn & al; 2012	RCT/Evaluate the Health Quality Partners program’s effect on mortality	Community based nurse care management	65 years of age and older with multimorbidity	Exclusive care management role; A network of primary care practices, eastern Pennsylvania, USA	Target eligible patients; visit at home when necessary; assess patient’s needs; develop a care plan; monitor, follow up and respond to change; support self management and behavior change; assess and promote patient adherence to treatment; reconcile errors/omissions with the GP; coordinate care with health and social care providers and family members; facilitate care transition; manage advance directives; document in patient records

Ekers & Wilson; 2008	Audit of symptom outcome and satisfaction/Examine the impact of interventions delivered in routine primary care clinics by nurses	Case management	Patients with moderate to severe depression	Case management with other primary care duties; General practitioner practices, the North East of England	Assess patient’s symptoms and risk factors; manage medication; support behavioral activation; discuss cases with psychiatric nurse and GP; respond to changes

Friedman & al; 2009	RCT/Report the impact of a primary care health promotion nurse intervention	Disease management	High risk patients with disability (or their caregiver) and recent significant (or expected) health care utilization	Exclusive disease management role; Home visits, counties in western New York, in West Virginia, and in Ohio, USA	Visit patient at home; assess their status; monitor medication list; provide education and support for self management and behavior change and maintenance; facilitate communication between patient and family and GP

Gabbay & al; 2013	RCT/Determine whether the addition of nurse case managers to usual care would result in improved outcomes	Case management	Diabetic patients at high risk for complications, in an underserved Hispanic population	Exclusive case management role; Primary care clinics within two health systems in Central Pennsylvania, USA	Monitor and follow-up; provide counseling and enhance motivation to change and medication adherence; engage in phone and email conversations with patient between visits when needed; make referrals to specialty services when necessary; discuss medication list with GP

Hudon & al; 2015 (same intervention as in Hudon 2018)	Qualitative study/Examine the experience of patients and their family members with care integration in a case management program by primary care RN	Case management	Frequent users of health care services who had chronic diseases	Case management role with other primary care duties; Family medicine groups, Province of Quebec, Canada	Assess patient’s needs and resources; develop a personalized care plan; involve patient in decision-making; provide education, counseling, self management support; build trust through interpersonal communication; improve transition, communication and coordination with and among healthcare and community partners

Jansen & al; 2011	RCT/Compare the effects of case management and usual care	Case management	Community-dwelling older adults with dementia symptoms and their informal caregivers	Exclusive case management role; Home visits, West-Friesland, the Netherlands.	Visit patient at home; assess patient and caregiver situation; formulate a care plan with them; provide them education; improve their social support; refer them to relevant healthcare professionals; monitor and follow-up; provide phone consultations; inform the GP; discuss cases with peers; document and share information

Katon et al; 2012 (same intervention as in Trehearne 2014)	RCT/Evaluate the cost-effectiveness of a collaborative treatment program	Collaborative care management	Patients with poorly controlled diabetes mellitus, CHD, or both and comorbid depression	Exclusive care management role; Primary care clinics of an integrated healthcare system, Washington State, USA.	Visit patient at home; identify clinical goals; develop individualized care plans; monitor, follow up and respond to change; provide patient education and support for self management; review and discuss cases with GP and specialists

Mastal & al; 2007	Case study/Explore innovative practices in two disability care coordination programs	Care management	Adults with severe and persistent mental illness who had developed type II diabetes	Care management role with leadership responsibilities; Home visits, RN is located in both the community mental health center and the primary care center, Vermont, USA	Visit patient at home; assess their health; accompany them in their trips out of their home if desired; provide education and self management support; link the mental health and primary care systems; interact with, educate and learn from mental health providers; serve as a resource to patient and providers

McNab & Gillespie; 2015 (same nurses’ role as in McNab &al; 2016)	Case study/Report on a program that aims to improve coordination and integration of services, and reduce unnecessary hospital readmissions	Integrated care	Older people with chronic and complex illness who are at risk of further exacerbation and/or hospitalisation	Exclusive role of liaison nurse; Within the larger Mount Druitt Community Health Centre; in homes; and at general practitioner practices, West of Sydney, Australia	Visit patient at home; identify their needs; initiate care planning; support self-management, provide a single point of contact and a familiar face for patient and teams; manage communication, case conferencing and care coordination between professionals involved in patient’s care; make referrals into health and social care

Meng & al; 2009	RCT/Evaluate the effect of a primary care nurse intervention on paid personal assistance use and expenditures	Disease management-health promotion	Community dwelling older people with disabilities and recent significant health care utilization	Exclusive role of disease management-health promotion; Home visits, New York, West Virginia, and Ohio, USA	Visit patient at home; co-create goals; collaborate with patient, GP and MDT to create care plans, support behavior change, medication management and treatment adherence; provide education and support for self management to patient and caregivers; facilitate transition and communication between the GP, patient and caregivers

Metzelthin & al; 2013	Mixed methods/Examine the implementation of the interdisciplinary care approach and participants experiences	Case management	Community-dwelling frail older people (> 70 years)	Case management role with other primary care duties; General practices, the south of the Netherlands	Contribute to patients targeting; visit patient at home; perform a comprehensive assessment; inform and cooperate with patient, caregivers and MDT for the formulation and execution of a treatment plan; monitor and follow up; facilitate the protocol implementation

Newcomer & al; 2004	RCT/Present patient outcomes after 12 months of participation to the preventive case management program	Case management	Older people with chronic condition	Exclusive case management role; Primary care medical groups within Sharp HealthCare of San Diego, California	Perform health risk screening and contribute to triage; formulate multidimensional care plan interventions; provide education; assist patient in preparing their appointments with the GP; monitor and facilitate treatment adherence; reconcile medication; provide referrals and assistance with services access; communicate with GP; facilitate health plan processes navigation; coordinate with the hospital discharge planners; monitor and adapt risk status

Roth & al; 2012	Study cohort/Examine Community Care Managers’ assessments and interventions compared with PCP care	Care management	The most vulnerable older adults enrolled in a community Special Needs Plan (based on patient’s categories of diagnoses and disease severity to predict medical expenditures)	Exclusive care management role; Evercare Special Needs Plan site located in central Florida, USA	Visit patient at home; assess their needs; develop a personalized care plan; promote preventive services; engage with GP; provide health education and caregiver support; coordinate services; facilitate transitions and communication between all parties including the patient

Ruikes & al; 2016	Cluster Controlled Trial/Evaluate the effectiveness of a general practitioner–led program integrating cure, care, and welfare for the prevention of functional decline	Case management	Community-dwelling frail older people (> 70 years)	Unclear*; General practices, Nijmegen, the Netherlands	Visit patient at home; involve them in setting goals based on the assessment of their needs; formulate individualized care plans; ensure patient acknowledges these plans; review medication with GP and pharmacist and document decisions in the care plan; maintain treatment contact with patient and caregivers; organize and participate to team meetings

Schraeder & al; 2008	Non-randomized comparison/Test the effectiveness of a management intervention on healthcare utilisation and cost	Case management	Chronically ill community dwelling older people at high-risk for mortality, functional decline, or increased health services use	Exclusive case management role; Primary care practice, East central Illinois (intervention); West central Illinois (comparison), USA	Visit patient at home; perform a comprehensive assessment and integrate it with the GP’s; develop a care plan with the GP; adjust it when necessary; monitor and follow up; provide patient education and support for self management and behavioral change; monitor the need for educational interventions; visit patient during transitional periods and provide ongoing monitoring and assessment; build a personal relationship with patient; communicate with and transfer information to GP and team; coordinate supportive services

Scrymgeour & al; 2013	Mixed methods/Assess the effectiveness of the Continuity of Cancer Care pilot	Care coordination	Patients with varying stages of cancer progression	Exclusive care coordination role; General practices in New Zealand	Provide emotional support, verbal guidance and support for self management to patient and family; include patient and family in care; respect patient’s values and beliefs; serve as a main point of contact available when needed; advocate on behalf of patient with other healthcare providers; link patient with GP, MDT and services

Spencer; 2019	Quasi-experimental uncontrolled before and after design involving a retrospective review/Evaluate a quality improvement project	Care coordination	Underserved, uninsured community with Type II diabetes	Exclusive care coordination role; Community Health in Partnership Services (CHIPS) clinic, St. Louis City, Missouri, USA	Form a meaningful relationship with patient and providers; clarify roles and responsibilities with patient; act as a resource for the team; assist patient and family completing the plan of care; document; provide technical support; make referrals when necessary

Suijker & al; 2016	Cluster Randomized Trial/Evaluate the effects of nurse-led multifactorial care to prevent disability	Care coordination	Patients aged 70 years and over at increased risk of functional decline	Exclusive care coordination role; General practices, north-west of the Netherlands	Visit patient at home; conduct a comprehensive assessment; co-construct care plan with patient and GP where roles are clearly defined; monitor and follow-up; collaborate with patient, GP and MDT

Thyrian & al; 2017	Cluster Randomized Trial/Test the effectiveness and safety of Dementia Care Management	Care management	Patients aged 70 years and over, living at home, and diagnosed as having dementia	Exclusive care management role; General practices, municipalities of Mecklenburg-Western Pomerania, Germany	Visit patient at home; conduct a comprehensive assessment; discuss intervention tasks with MDT; inform the GP and establish an individualized care plan with them; cooperate with the caregiver, GP, and health and social care providers; manage treatment, care and medication; provide caregiver support and education; monitor and follow-up

Unutzer & al; 2008	Prospective pilot study (mixed methods)/Test the feasibility of and generate preliminary evidence for the efficacy of a care management program	Care management	Patients aged 60 years and over with depression and osteoarthritis pain	Exclusive care management care; Primary care clinics, University of Washington’s Practice Network, Washington, USA	Conduct a comprehensive assessment; manage medication; support activation; provide education and support for self management; coordinate with GP and specialist; monitor and follow-up and respond to change

Van Leeuwen & al; 2015	Economic evaluation and a stepped-wedge cluster-RCT/Evaluate the cost-effectiveness of the Geriatric Care Model	Integrated care	Frail persons aged 65 years and over at increased risk of functional decline	Exclusive care coordination role; Primary care practices, two regions in the Netherlands	Visit patient at home; conduct a comprehensive assessment; establish a care plan with GP; discuss it with patient; inform and involve patient in decision making; document; monitor and follow up; coordinate care with MDT and community network professionals

Yuille; 2015	Qualitative descriptive study/Identify, from the perspective of RNs, the strengths, gaps, barriers, and opportunities for optimizing nursing roles in the delivery of cancer survivorship care in primary settings	Chronic disease management	Patients with varying stages of cancer progression	Chronic disease management with other primary care duties; Local Health Integration Networks across Ontario, Canada.	Establish a long standing and trusting relationship with patient; serve as their “go to” person; provide emotional support; provide education and support for self management; manage care; assist patient and family navigate through the healthcare system; arrange and coordinate services for them; facilitate access to community resources; facilitate transitions.


*Although the care coordination role is clearly described, it is unclear if the nurses are employed to carry out this role exclusively or combine care coordination with other primary care duties. Legend: CHD = Coronary heart disease GP = General practitioner MDT = Multidisciplinary teams RN = Registered nurses

### Care coordination interventions

Authors used several terms to describe care coordination interventions. The included papers reported on case management [34,43,44,45,46,47,48,49,50,51,52], care management [33,53,54,55,56,57,58], disease management [59,60,61], guided care [32,62,63,64,65], integrated care [66,67,68], care coordination [69,70,71], and nurse-led care interventions [72,73]. Definitions of these interventions were not always provided and, as reported in ***Table 1***, we noted variations in care coordination activities within the same type of intervention. For instance, within case management interventions, a comprehensive initial assessment of patient and family needs and goals was explicitly mentioned in several, but not all, studies [46,47,48,49,50,51,52].

### Complex target patient populations

The target patient populations identified in the included studies were also diverse. We have gathered them into seven categories according to their common areas of vulnerability as presented in the “Complex health and social care needs” model (***Figure 1***) above, as well as their frequent use of health care services. ***Table 2*** displays this categorization.

**Table 2:** Categories of identified patient populations with complex health and social needs.


CATEGORIES OF PATIENT POPULATIONS WITH COMPLEX NEEDS	IDENTIFIED TARGET POPULATIONS IN INCLUDED STUDIES

1. Patients with severe mental illness	Patients with moderate to severe depression [43,52]

2. Patients with common or severe mental illness + chronic condition(s)	Adults with severe and persistent mental illness who had developed type II diabetes [55] Patients with poorly controlled diabetes mellitus, coronary heart disease or both and comorbid depression [53] Patients aged 60 years and over with depression and osteoarthritis pain [58]

3. Patients with social vulnerability + chronic condition	Low-income, ethnically diverse populations at elevated risk of cardiovascular disease events, including those with existing coronary heart disease or diabetes [44] Diabetic patients at high risk for complications, in an underserved Hispanic population [45] Underserved, uninsured community with type II diabetes [70]

4. Older people with physical, cognitive, psychosocial or psychiatric vulnerabilities (+ significant healthcare use)	Community-dwelling frail people aged 60 and older [72] Community-dwelling frail older people (> 70 years) [49,51] Patients aged 70 years and over at increased risk of functional decline [71] Frail persons aged 65 years and over at increased risk of functional decline [68] Older patients with multimorbidity and functional disability and who had generated high insurance expenditures for health care [32] Community dwelling older people with disabilities and recent significant health care utilization [60] High risk patients with disability (or their caregiver) and recent significant (or expected) health care utilization [59] The most vulnerable older adults (based on patient’s categories of diagnoses and disease severity to predict medical expenditures) [56] Chronically ill community dwelling older people at high-risk for mortality, functional decline, or increased health services use [50]

5. Old age people + chronic condition(s)	Older people with chronic and complex illness who are at risk of further exacerbation and/or hospitalisation [66] 65 years of age and older with multimorbidity [33] Community-dwelling older adults with dementia symptoms and their informal caregivers [47] Patients aged 70 years and over, living at home, and diagnosed as having dementia [57] Older people with chronic conditions [48]

6. Patients living a transition to end-of life/palliative stage	Patients with varying stages of cancer progression [61,69]

7. High costs and frequent users of health care services	Frequent users of healthcare services who had chronic diseases [46]


### Care coordination activities

In addition to the variations in care coordination activities within the same type of intervention, we noted a variation when we tried to compare care coordination activities according to target patient populations. For instance, within the category of older patients with multiple vulnerabilities and significant use of healthcare services (category 4), facilitating care transitions was not mentioned in all studies, although present in several studies [32,50,56,60].

**Figure 3:**
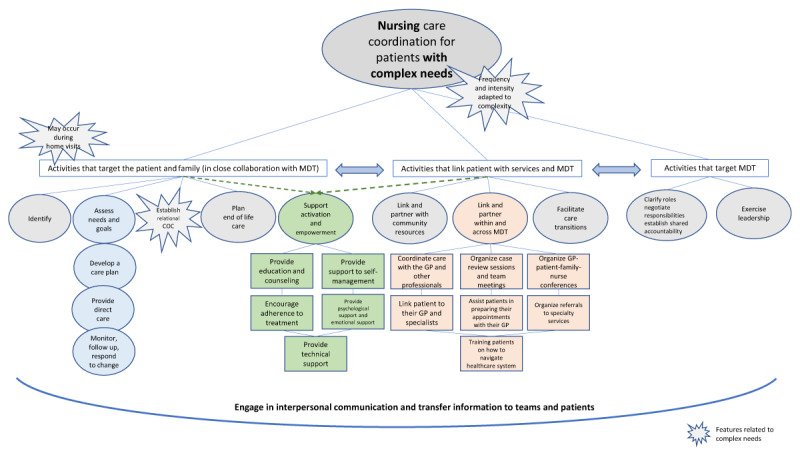
A model of nursing care coordination activities for patients with complex needs. Legend: COC = Continuity of care GP = General practitioner MDT = Multidisciplinary teams (including health and social care professionals).

We were able to synthesize overall nursing care coordination activities into three categories: activities targeting the patient, family and caregivers; those targeting health and social care professionals and services; and those bringing together patient and professionals. One last category was found to be cross-cutting, supporting and enhancing every other activity, namely interpersonal communication and information transfer (***Figure 3***). In the following section, we present the four categories and their respective activities; a more detailed description of these activities is provided in Appendix 2.

#### Care coordination activities targeting the patient, family and caregivers

– Identify patients who will benefit most from the intervention: in close collaboration with the general practitioner, care coordinator nurses contribute to the identification of patients with complex health and social care needs and/or high expenditures for health care and invite them to receive the intervention [33,51].
– Assess comprehensive patient and family needs and goals [32,33,34,46,47,48,49,50,51,52,53,55,56,57,58,59,60,66,68,71,72], including caregivers’ burden [32,47,73].
– Develop a tailor-made care plan with the patient and personalize it to align with their unique individual circumstances [32,33,34,46,47,48,49,50,53,56,57,60,66,68,71,72]. This plan provides every health and social care professional involved with a summary of the patient’s status and plans [46,51,57,66]. It is reviewed and updated as needed [49,50,58,68,71]. In some cases, it is written in lay language and displayed prominently in the patient’s home [32]. Finally, as part of this care planning, nurses educated the patient about the care coordination efforts being made to improve their quality of care and what the patient’s responsibilities were [70].
– Provide care directly: nurses follow explicit guidelines and protocols for disease risk reduction [44], and conduct health status and physiologic monitoring (i.e. blood pressure and blood glucose) [44,74]. Other nursing tasks include carrying out basic screening (i.e. cancer screening) and managing symptoms as well as episodic illness and concurrent chronic diseases [61].
– Monitor, follow up, and respond to change: monitoring includes symptoms, clinical results, current medications, errors or omissions, adverse effects, and adherence to therapeutic plan [32,33,43,45,47,48,50,51,52,53,57,58,59,68,71,72]; but also, emergency department visits, hospital admissions, or any other encounters that would change the risk status and trigger an intervention [48,50].
– Establish relational continuity of care by building an ongoing, personal and meaningful relationship of mutual trust with the patient over time [46,50,61,66,69,70], advocating for them [69], and serving as their main point of contact and their “go to” person at all times [32,34,45,47,61,66,67,69].
– Plan end of life care: nurses identify the presence of advance directives, inform the patient regarding their right to state their preferences for care at the end of life, and assist the patient and family with planning end of life options [33]. They also provide ongoing emotional support to the patient and family [61].
– Supporting patient activation, engagement and empowerment requires a collaborative relationship between nurses and patients and their families, the involvement of the entire care team in planning, carrying out, and following up on patient care, and planning a coherent and continuous set of support methods [69]. These activities include enabling patients to be involved in treatment and diagnostic choices, to collaborate with providers, and to navigate the healthcare system and community resources [75,76,77].

Thus, they are embedded in many of the other activities described earlier, such as assessing patients’ individual needs and goals, facilitating their participation in care, involving them in the development of their own care plan, serving as their main point of contact and advocating for them, and helping them state their individual preferences for care at the end of life. Patients’ activation and empowerment also include linking patients with health and social care teams in order to enable engaged participation and shared decision-making as described further below.

We have also identified a list of additional performed activities aimed at supporting patient and family activation and empowerment, frequently using motivational interviewing principles and cognitive and behavioural strategies [32,33,34,45,46,53,59]. These include the provision of:

- individualized patient and family education and counseling [32,33,34,44,45,46,47,48,50,52,55,56,57,58,59,60,61,68,69];
- support for self-management [32,33,34,43,44,46,48,53,55,58,60,61,66,69,72];
- support for adherence to treatment [32,33,43,44,45,46,48,58];
- psychological and emotional support [32,33,34,43,46,47,55,56,57,61,67,69];
- technical support for accurate monitoring of biological parameters (i.e. glucose or blood pressure) [53,55], or for carrying out administrative tasks [70].

These activities are not sequential but rather iterative. Typically, a comprehensive assessment is needed in order to identify patients who would benefit from a care coordination intervention; in turn, patients identified as target beneficiaries require a comprehensive assessment of their needs and goals in order to develop their care plan.

Finally, we were able to identify three main features of the activities targeting the patient and family: 1) they may occur during home visits; 2) they are undertaken in close collaboration with the general practitioner and health and social care teams. This is particularly relevant when targeting eligible patients and care planning; 3) they are complementary to and do not replace the general practitioner’s activities. Nurses organized care that the general practitioner was not able to organize due to lack of time or because they did not have the knowledge of services available [51,66,67], and seemed to be very effective in areas that are less extensively addressed by the general practitioner such as complex assessments of function and social support [56] or self-management support [54]. Overall, collaboration between nurses and general practitioners resulted in a broader assessment of a patient’s health and the development of more comprehensive care plans [50] thus, improving health management.

#### Care coordination activities targeting health and social care professionals and services

– Clarify roles, negotiate responsibilities and establish shared accountability: care coordinator nurses explain their role to other professionals [32,51] and, through the development of the care plan, discuss and specify all actions expected from each participant and discipline [51,57,71]. They ensure accountability by systematically reviewing and discussing cases with the general practitioner and other team members, and jointly deciding on appropriate actions to take [32,33,51,52,53,57].
– Exercise leadership: nurses build relationships and personal credibility with other professionals [55]. They provide local knowledge and a single point of contact and a familiar face for health and social care providers [66,67]. They serve as a resource to the team [55,70] and facilitate the implementation of an interdisciplinary care approach with their organizational and communication skills and empathic capacity [51,67].

#### Care coordination activities that link the patient and family with health and social care professionals and services

– Link and partner with community resources (outside the healthcare system): nurses coordinate, arrange and monitor access to community resources and social care tailored to patients’ specific needs (i.e. public housing, meal services, financial assistance services, smoking cessation, self-management support course led by trained lay people, etc.) [32,33,44,46,48,50,60,61,66,69,72]. They also provide a guidebook with available social and welfare services [47].
– Link and partner within and across multidisciplinary healthcare teams. This includes:
  - coordinating patient care with the general practitioner and other healthcare professionals [32,33,34,43,45,46,47,48,50,51,55,56,58,71];
  - organizing case review sessions and team meetings to discuss patient specific situations and innovations in care [49,50,52,53,55,57,66,68], communicate changes in treatment plan, discuss medication management [33,48,49,60], or discuss questions the patient had but were uncomfortable to ask to their general practitioner [50];
  - organizing general practitioner-patient-family-nurse meetings to facilitate communication [59,60];
  - linking patients to their general practitioner and specialists [32,33,44,46,47,55,69];
  - assisting patients in preparing their appointments with their general practitioner [48];
  - organizing referrals to specialty services when needed [43,44,45,47,48,69,70,71]; and
  - training patients on how to identify and navigate the healthcare system [48].
– Facilitate care transitions consists of smoothing the patient’s path between all services and care providers with a focus on transitions through hospitals [32,33,56,60,61]. Care coordinator nurses coordinate with but do not replace the hospital discharge planning professionals and provide them with information on home environment and safety and any caregiver issues that may affect discharge planning [48]. Nurses perform ongoing monitoring and assessment tasks during transitions [50], adjust patient care plans to meet current needs [33,50] and keep the general practitioner informed of the patient’s current status [32].

#### Cross-cutting activities related to interpersonal communication and information transfer

– Engage in open and honest communication with patients about their health and social situation: honest communication seems to support every other activity with the patient and family, starting with a comprehensive assessment of their needs and goals [46], the discussion of questions that they were uncomfortable asking their general practitioner (linking them with other professionals) [50], and the establishment of relational continuity that fosters a trusting and meaningful relationship [67].
– Engage in interpersonal communication with health and social care professionals: again, every single activity that nurses undertake with and for professionals seems to be supported by interpersonal communication. This is particularly relevant to clarifying roles and responsibilities [32,51,71], establishing shared accountability [32,33,50,51,52,53,57], providing local knowledge [47] and facilitating the implementation of an interdisciplinary care approach [51,67,70].
– Transfer information: nurse care coordinators communicate care plan letters and other relevant information to patients and families [33,50]. They transfer information to the general practitioner and other health service providers about issues identified in the screening, treatment, service provided to the patient, clinical evolution, etc. [43,46,48,50,51,57,60,66]. They also document this information and provide timely updates [32,33,43,46,47,49,66,68,70].

## Discussion

Nurses are playing an increasingly central role in care coordination. However, no previous model has provided an in-depth analysis of this role for patients with complex health and social care needs in primary care.

Our conceptual framework of care coordination provides an extensive description of each care coordination activity (or domain). This new information could constitute a valuable contribution that facilitates the design and implementation of care coordination interventions for patients with complex needs.

Furthermore, our synthesis of care coordination interventions is based on empirical studies and with a focus on the way these interventions are operationalized. We propose a pragmatic model of care coordination for patients with complex needs that would be close to the field reality of health and social care professionals.

### Summary of findings

Our results showed the multitude of interventions used to coordinate care around patients with complex health and social care needs in primary health care. This heterogeneity was expected and confirms the fact that care coordination can be operationalized in different ways. Care coordination interventions also differ on the basis of their context, the population of interest, and the goals of the program [78].

The care coordination model that we propose should not be regarded as a manual to be followed but rather as a tool to guide professionals and decision makers tailoring their own intervention based on the needs of their target patient population as well as their contextual and environmental realities. The success and failure of integrated care interventions, including care coordination, have always been context dependent [79,80].

Our findings also confirmed the fact that the care coordination role may be undertaken exclusively or combined with other primary care duties. In fact, nurses seem to perform the same care coordination activities independently of their employment arrangement. We hypothesize, though, that what may be different is the caseload of nurses and, again, the intensity of their support. Goodman (2010) showed that exclusive case managers provided greater input to their patients and higher contact time per month than did nurses who undertook other duties concurrently [81]. However, the authors also pointed out the increased cost of exclusive case management. Therefore, healthcare organizations are faced with the challenge of finding employment models that can ensure balance between efficiency and intensive care coordination for target patient populations or individuals.

This review shed light on critical components of care coordination that are specific to patients with complex needs. Firstly, although the care coordination model may seem to include the same activities for patients with long-term conditions without particular complexity, the main difference is the greater frequency and intensity of activities when caring for patients with complex needs. Several studies highlighted the need for care coordination to be targeted according to the varying intensity of needs [19,81]. Leutz (1999) and Kodner (2002) addressed the question of “who needs what level of integration” [82,83]. The authors suggested that different levels of integration should exist for patients according to their needs, their capacity for self-direction, and the specific challenges they face in obtaining appropriate care [82]. Our findings illustrate how complex patient groups would require a broader span, more intense, and more thoroughgoing interventions. Garcia et al. (2018) conceptualized a spectrum of care management need that ranges from complex patients who have sufficient skills and resources thus “not needing intensive care management” to “patients needing more than intensive care management” [40]. For the first group, patient navigation, a less intensive intervention, may address their needs effectively by reducing barriers and bridging gaps in service [84]. The patient navigation role may indeed be played by nurses and social workers or by lay persons trained adequately [85]. Patients at the other end of the spectrum are those requiring more specialized interventions beyond those offered by care management [40]. Between these two extremes of the spectrum, there are the patient populations identified in our review and for whom a comprehensive, intensive care coordination intervention, by a professional, is necessary. In fact, the included studies described patient navigation as one component of a wider intervention.

Primary care registered nurses provide a broad range of services for patients with long-term conditions without particular complexity. These include chronic disease prevention and management, medication management, health education and therapeutic interventions [86]. These activities seem to be very present when coordinating care for patients with complex needs. Indeed, our findings highlighted activities related to their clinical skills such as conducting a comprehensive assessment including for medical needs and goals; careful monitoring of patient symptoms; early identification of exacerbations; medication reconciliation and early identification of adverse effects; providing patient education that includes self-management of their medical conditions and recognizing alarming symptoms, as well as providing direct evidence-based care. Previous results indicate that when nurses are an integral part of direct care through the management of the interdisciplinary team, programs have a great opportunity to improve quality of care and clinical outcomes, and reduce beneficiaries’ need for hospitalization [87,88].

Secondly, the establishment of a relational continuity of care with the patient and family also emerged as a critical component of care coordination for patients with complex needs. In addition to the intensity of support, our results emphasize the availability of the care coordinator out of hours and when urgent issues arise, which leads to the development of a great relational continuity of care. In fact, for patients with complex needs, integrated care often means a single point of entry and a personal contact with a designated care coordinator [89]. Our findings show how nurses become patients’ “go to” person at all times. Studies showed that relational continuity improved preventive care, reduced hospitalization, enhanced adherence to treatment, and increased satisfaction with care [90,91].

Finally, home visits seem to be particularly important when coordinating the care of patients with particular needs or certain complex needs. This is mainly in response to the target patient populations’ vulnerability and sometimes functional decline [92], but also because it allows nurses to gain more insight into the patient’s living environment, including safety issues [33,48] and caregiver burden [93], which are crucial to care planning. Nevertheless, home visits should not be regarded as an isolated activity, but rather as a feature of a comprehensive care coordination intervention whose first step is to know the patient and caregiver in order to identify what care or service they need [94].

### Implications of the results on research and practice

It is widely recognized that complex interventions are often not delivered or adhered to as intended [49]. Yet, studies included in our review rarely reported on their assessment of intervention fidelity. Furthermore, we do not know if nurses performed one activity more than another, or whether they needed capacity building for the least performed activities. Future research should address these two areas since answers might explain, at least partly, the limited efficacy of some interventions.

Also, it would be interesting to attempt and establish a classification of activities according to their value, i.e. efficacy versus time and resource consumption.

Our scoping review did not aim to compare the nurses’ role in care coordination to that of another professional. Future research could highlight the specificity of each professional and their added value in the coordination of care.

In practice, the co-location of health and social care professionals has gained considerable attention over the last few years for what it offers in terms of facilitating multidisciplinary teamwork and the delivery of integrated care [81]. Our findings reaffirm the fact that no single professional has the ability to complete the task independently, and that “close” collaboration between health and social care services is particularly important for eective care coordination. This review could therefore constitute an additional argument in favour of the staff co-location.

Finally, recognizing care coordination in practice requires defining the work and purposefully examining when, where and how it is happening [87]. This synthesis would support the development of a system to document nurses’ care coordination activities as a way of ensuring appropriate financial and societal recognition of their contribution to high quality, efficient and integrated care.

### Strengths and limitations

To our knowledge, this is the first study to synthesize nursing care coordination activities for patients with complex needs in primary healthcare. We were able to propose an exhaustive model of care coordination, as well as a comprehensive description of patients’ complex health and social care needs. Another major strength would be the validation of our findings by a patient-research-partner.

As for limitations, this scoping review did not provide a comparison between care coordination interventions or their efficacy. The complexity and heterogeneity of interventions represent a real challenge for comparison and necessitate the use of research designs other than a scoping review. Another limitation could be related to the fact that our findings are based on the description of activities as provided in included studies. These descriptions were sometimes poor or lacked important elements, so we may have missed details or features of some activities. However, to mitigate the missing information, we examined all articles reporting on the same intervention.

## Conclusion

A multitude of interventions are used to coordinate the care of patients with complex health and social care needs in primary health care. Despite this heterogeneity, they share a commonality in that they involve a great frequency and intensity of care coordination activities related to the complex needs of the target patient populations. Care coordinators establish a great continuity of care with these patients due to their availability outside of regular hours and when urgent issues arise. As complexity grows, efforts must be directed towards enabling the primary healthcare level to effectively play its substantial role in care coordination. This includes finding primary care employment models that would facilitate multidisciplinary teamwork and the delivery of integrated care, and guarantee the delivery of intensive yet efficient coordinated care.

## Additional Files

The additional files for this article can be found as follows:

10.5334/ijic.5518.s1 Appendix 1. Search strategy.

10.5334/ijic.5518.s2 Appendix 2. Care Coordination activities and their description.
