# Supplementary material for: Nursing Care Coordination for Patients with Complex Needs in Primary Healthcare: A Scoping Review
Source: Int J Integr Care. 2021 Mar 19;21(1):16. doi: 10.5334/ijic.5518 (PMC7977020; doi:10.5334/ijic.5518)
Supplement: Appendix 1. — Search strategy. [file ijic-21-1-5518-s1.pdf]

Appendix 1 : Search strategy

Interface: EBSCOhost
Databases: CINAHL Plus with full text; Medline with full text
Date: October 9th, 2019
Hits : 2264

≠1	TI ("case management" OR "care management" OR "disease* management" OR "patient navigation" OR "integrated care" OR "care coordinat*" OR "patient-centered medical home" OR "Delivery of Health Care, Integrated") OR AB ("case management" OR "care management" OR "disease* management" OR "patient navigation" OR "integrated care" OR "care coordinat*" OR "patient-centered medical home" OR "Delivery of Health Care, Integrated") OR SU ("case management" OR "care management" OR "disease* management" OR "patient navigation" OR "integrated care" OR "care coordinat*" OR "patient-centered medical home" OR "Delivery of Health Care, Integrated")
≠2	TI ("primary health care" OR "primary care" OR "primary healthcare") OR AB ("primary health care" OR "primary care" OR "primary healthcare") OR SU ("primary health care" OR "primary care" OR "primary healthcare")
≠3	TI nurs* OR AB nurs* OR SU nurs*
≠4	1 AND 2 AND 3
Limiters	Language: English, French<br>Research article<br>Publication type: case study, clinical trial, journal article, nursing intervention, randomized controlled trial, research<br>Search modes: find all my search terms

Databases: Scopus
Date: October 17th, 2019
Hits: 1753

(TITLE-ABS-KEY ("case management" OR "care management" OR "disease* management" OR "patient navigation" OR "integrated care" OR "care coordinat*" OR "patient-centered medical home" OR "Delivery of Health Care,Integrated")<br><br>AND<br><br>TITLE-ABS-KEY ("primary health care" OR "primary care" OR "primary healthcare")<br><br>AND<br><br>TITLE-ABS-KEY ("nurs*")) AND (LIMIT-TO (PUBSTAGE , "final")) AND (LIMIT-TO (DOCTYPE , "ar"))<br><br>AND<br><br>(LIMIT-TO (LANGUAGE , "English") OR LIMIT-TO (LANGUAGE , "French"))

Databases: ProQuest (dissertations and thesis)
Date: October 8th, 2019
Hits: 346

(ti("case management" OR "care management" OR "disease* management" OR "patient navigation" OR "integrated care" OR "care coordinat*" OR "patient-centered medical home" OR "Delivery of Health Care, Integrated") OR ab("case management" OR "care management" OR "disease* management" OR "patient navigation" OR "integrated care" OR "care coordinat*" OR "patient-centered medical home" OR "Delivery of Health Care, Integrated") OR su("case management" OR "care management" OR "disease* management" OR "patient navigation" OR "integrated care" OR "care coordinat*" OR "patient-centered medical home" OR "Delivery of Health Care, Integrated")) <br>AND <br>(ti("primary health care" OR "primary care" OR "primary healthcare") OR ab("primary health care" OR "primary care" OR "primary healthcare") OR su("primary health care" OR "primary care" OR "primary healthcare")) <br>AND <br>(ti("nurs*") OR ab("nurs*") OR su("nurs*")) <br>AND <br>la.exact("ENG" OR "FRE")
