# Supplementary material for: Nursing Care Coordination for Patients with Complex Needs in Primary Healthcare: A Scoping Review
Source: Int J Integr Care. 2021 Mar 19;21(1):16. doi: 10.5334/ijic.5518 (PMC7977020; doi:10.5334/ijic.5518)
Supplement: Appendix 2. — Care Coordination activities and their description. [file ijic-21-1-5518-s2.pdf]

Appendix 2: Care Coordination activities and their description

**Activities that target the patient, family and caregivers (in close collaboration with the MDT)**	**Description**
Identify patients who will benefit most from the intervention	• Target and identify patients with chronic diseases, complex health and social care needs, and/or high expenditures for healthcare<br>• Classify patients into risk strata and include those scoring at or above the high-risk stratum<br><br>Strategies that aim at identifying patients include:<br>○ Case-finding based on the judgement of the nurse and GP<br>○ Use of screening instruments (interview-based or self-reporting questionnaire)<br>○ Use of medical records to target frequent users<br><br>Stratification is usually performed in close collaboration with the GP
Assess comprehensive patient and family needs and goals	• Assess:<br>○ Patient's medical, functional, cognitive, behavioral, affective, psychosocial, nutritional, and environmental needs and resources<br>○ Existing problems and risk factors (i.e. fall risk, loneliness, developing disability)<br>○ Patient's healthcare goals<br>○ The family needs and issues<br>○ The caregiver capacity and burdens<br><br>• Use validated and standardized tools<br>• Conduct home visits if necessary<br>• Schedule follow up assessments as needed

Develop a tailor-made care plan	• Develop and discuss care plan with the active involvement of the GP, patient, and caregiver<br>• Personalize it to align with the unique circumstances of the individual patient (preferences, values, priorities, intentions, motivational readiness)<br>• Involve patients in decision-making and establish a cooperation in which a learning process begins leading to new insights and possibly new goals and actions<br>• In few contexts, provide innovative and proactive care, with an evidence-based approach, based on patients' needs<br>• Educate the patient about the care coordination efforts being made to improve their quality of care and what the patient responsibilities were<br><br>The CP:<br>○ Is proactive<br>○ Involves goals, strategies and responsibilities that fit patients' needs<br>○ Is based on the information gained from the comprehensive assessment (and takes findings from risk assessments into consideration)<br>○ Includes individualized health and social service plans with the collaboration of the health and community partners<br>○ Is derived from evidence-based care plans<br>○ Identifies specific predefined trigger conditions and a subsequent intervention task (i.e. when the patient is to call the nurse care manager, the GP, or emergency services)<br>○ Is integrated into the practice's digital patient information system, when technically possible<br>○ Provides all involved healthcare professionals with a concise summary of the patient's status and goals<br>○ Is reviewed and updated as needed during follow-up visits<br>○ Is written in lay language and displayed prominently in the patient's home
Monitor, follow up, and respond to change	• Systematically and proactively **monitor**: (either by telephone or in person)<br>○ Symptoms, clinical results, current medications and possible errors/omissions, adherence to the therapeutic plan, the possible adverse effects of the treatment

	○ ED visits, hospital and nursing home admissions, home health visits, and durable medical equipment orders from the health plan's data systems. Such encounters would change the risk status and trigger a telephone contact<br><br>• Conduct **<u>follow up</u>** visits regularly (either by telephone or in person) in order to:<br>○ Evaluate the achievement of goals, the implementation of strategies in daily life and the need for support in the following period<br>○ Formulate a maintenance plan for follow-up with the primary care team<br>○ Check whether patients were due for complications screening<br>○ Remind them of follow-up specialist visits when they were due<br><br>• **<u>Respond to change</u>**:<br>○ Identify new or worsening conditions or symptoms early, and facilitate timely interventions (to prevent exacerbation, hospital admissions, use of ED). Interventions could be relative to disease management, functional ability, medication problems, financial and legal assistance, or referrals to social and community services<br><br>Changes are also addressed on several key moments: following patients' hospital discharge, after their visits to the GP and specialists, and following ED visits
Establish relational continuity of care	• Build an ongoing personal and meaningful relationship of mutual trust with patients over time<br>• Advocate for patients with other health providers<br>• Develop their skills in therapeutic communication, basic counseling, and listening<br>• Serve as the main point of contact, the "go to" person by being:<br>○ Directly accessible by telephone and email to the patient and caregiver for questions and concerns<br>○ Available for patients between visits and for urgent care when needed<br>○ Informed of the patient's ED visits and hospitalization

<table>
<tr><td>Plan end of life care</td><td>Identify the presence of current advance directives (durable power of attorney for health care decisions, and living will) when relevant
Provide patients education regarding their right to self-determination and preferences for choosing a decision maker and to designate their individual preferences for care at the end of life
Anticipate patients’ future care needs and assist patients and families with planning to meet those needs – treatment, end of life options, living situation, etc.
They also develop a long-standing relation with patients and family members and provide them ongoing emotional support.</td></tr>
<tr><td>Support patient and family activation and empowerment</td><td><u>Enable patients to</u> be involved in treatment and diagnostic choices, to collaborate with providers, and to navigate healthcare system and community resources.
<u>Linking patients with health and social care teams</u> in order to enable their engaged participation and a shared decision making

<u>Use motivational interviewing principles and appropriate cognitive and behavioral strategies</u> (behavioral activation, problem solving, etc.) <u>to</u>:
Identify patient preferences and facilitate the patient’s participation in care
Continually assess patients’ motivational stage for behavior change
Assist the patient in developing and maintaining healthy behaviours: physical activity, nutrition, weight management, stress reduction, medication adherence, etc.
Reinforce adherence to the action plan
Provide individualized patient and family <u>education and counseling</u>:
<ul style="list-style-type:circle">
Develop a personalised, comprehensive, structured curriculum for disease specific education, disease prevention, health promotion, lifestyle and behavior change, medication management, and self-management
Provide this education one to one, or in a small group of participants</td></tr>
</table>

	○ Organize family meetings aimed at educating relatives, improving social support and relieving the primary caregiver<br>○ Provide customised instructional materials to accommodate each patient's health literacy and ability; (i.e. booklets, easily read newsletters, etc.)<br>○ Regularly assess the need for educational interventions. Changes in health status, frequent disease exacerbation, inability to perform ADL or IADL, etc. are considered as events that prompt this need<br><br>• Provide support to **self-management**:<br>○ Give practical advice on non-specific self-management strategies (regarding social and family relationships, unjustified self-criticism and self-esteem)<br>○ Promote patient's self-efficacy (i.e. by referring them to a free, local, self-management course led by trained lay people)<br>○ Encourage family or friends to become active in the therapeutic process and involved in the decision-making process<br>○ Discuss self-management techniques and procedures at case conferences where the patient is also present<br>○ Educate and support caregivers: initial assessment, a free self-management course, regular support group meetings (covering techniques to deal with issues such as frustration, fatigue, pain, and isolation; medication management; effective communication; etc), and ad hoc telephone consultation<br>○ Use a robust health education planning model (i.e. PRECEDE) as the organizing framework for the application of health behavior change strategies<br><br>• Encourage **adherence to treatment**:<br>○ Assess whether the patient had been able to initiate and continue treatment<br>○ Identify root causes for non-adherence<br>○ Utilize collaborative problem solving to address barriers<br><br>• Provide **psychological and emotional support**:<br>○ Assess the factors that are contributing to stress and identify the resources and techniques to manage stress

	○ Accompany individuals with mental illness on food shopping trips, walks, to healthcare appointments, if desired, to facilitate exercise and socialization<br>○ Make regular phone calls to check on patients and listen to them<br><br>• Provide **technical support**:<br>○ Complete applications for patient assistance programs and insurance companies<br>○ Facilitate transportation, coordinating with caregiver to accompany patients to the clinic<br>○ Assist in accurate glucose monitoring, …
**Activities that target health and social care professionals and services**	**Description**
Clarify roles, negotiate responsibilities, establish shared accountability	• Explain their role, do not usurp the duties of other professionals but instead provide each with current information<br>• Through the development of the care plan, discuss and specify all actions expected from each participant (including the patient) and discipline; overlapping tasks are minimized by formulating an integrated treatment plan and by involving collaborative goals<br>• Discuss problems with the GP, report new or worsening symptoms, abnormal findings, whether additional assessment by healthcare professionals is needed<br>• During case review sessions, discuss their problematic caseloads with GP, specialist, and other relevant healthcare providers. This allows taking immediate actions if needed and ensure accountability for follow-up to guideline-level disease management and achieving clinical goals
Exercise leadership	• Build relationships and personal credibility with health and social care providers<br>• Serve as a resource, provide local knowledge, a single point of contact and a familiar face for health and social care providers

	• Facilitate the implementation of an interdisciplinary care approach with their good organization and communication skills, and empathic capacity<br>• Improve communication, multidisciplinary involvement and teamwork in case conferencing and care planning<br>• Assess the need for evidence-based tools to assist the healthcare team developing care plans and effectively managing their patients' care
**Activities that link the patient and family with health and social care professionals and services**	**Description**
Link and partner with community resources (outside the healthcare system)	• Help identifying and arranging, and monitor access to community resources and social care (i.e. public housing, transportation or meals services, local Alzheimer's Association, Agency on Ageing, financial assistance services, pharmacy support programs, smoking cessation programs, self management support course led by trained lay people, memory groups, etc.)<br>• Provide a guidebook with available social and welfare services
Link and partner within and across multidisciplinary care teams	• **Coordinate care** between patients and family members and all care teams including (but not restricted to) the GP, specialists, hospital personnel, EDs, rehabilitation facilities, nursing homes, and pharmacies**:**<br>• Provide a brief synthesis of cases and offer suggestions for care changes to the GP<br>• Organize case review sessions with the MDT to discuss cases and innovations in care<br>• Communicate changes in treatment plan, or in-home environment and safety, discuss medication management, etc. during team meetings<br>• Discuss questions the patient had but were uncomfortable asking their physician, so all insights were shared by the team<br>• Organize physician-patient-family-nurse conferences to facilitate communication

	• Assist patients in preparing for their appointments through the provision of questions and issues to take up with their physicians<br>• Contact the GP and/or specialists to reconcile the medication list for proper dosing and frequency of administration in case of polypharmacy problems<br>• Act as a resource and liaison between primary care and specialized services<br>• Ensure continuity with any care for frequent physical morbidities<br>• Organize referrals to specialized nurses, and other healthcare professionals when needed (dietitian, podiatry, physical therapist, wound and ostomy care clinics, etc.)<br>• Organize patients and family members training in how to identify and navigate healthcare system<br>• Provide counseling and assistance with facility placement, hospice enrollment, and psychiatric evaluation when needed
Facilitate care transition	• Smooth the patient’s path between all sites and providers of care, focusing on transitions through hospitals<br>• Intensify visits when the patient is admitted/discharged from hospital, or any other healthcare institution<br>• Visit patients during their stays in institutions (visit to ED or admission to a hospital)<br>• Perform timely assessment to ensure safe and well coordinated care transitions<br>• Plan and execute follow-up: adjust patient care plans to meet current needs and help reduce exacerbations in health conditions<br>• Coordinate with but do not replace the hospital discharge planning medical groups<br>• Provide information on home environment and safety and caregiver issues that may affect the discharge planning arrangements<br>• Keep the GP informed of the patient’s current status

**Cross-cutting activities that support and enhance every other activity**	**Description**
Engage in interpersonal communication with patients and health and social care professionals	• Engage in open and honest communication with patients about their health and social situations<br><br>• Engage in Interpersonal communication with health and social care professionals through informal office conversations
Transfer information	• Communicate information to the GP and other health service providers about issues identified in the screening, treatment, service provided to the patient in the intervention, clinical evolution, unresolved problems including medication or appointment adherence difficulties, changes in patients' circumstances (admissions to hospital)<br>• Communicate care plan letters to patients and families<br><br>Information transfer to teams and patients may occur through a wide variety of channels: electronic mails, reports, written notes and summaries, fax, telephone, voice mail, or care plan letters<br><br>• **<u>Document and update</u>:**<br>• Integrate information into the patient's EHR, an information portal, or the patient's chart record<br>• Perform timely update notably through continuous monitoring, but also for completed action plan items (with the date this item was completed.

Legend:

ADL: Activities of daily living
CP: Care plan
ED: Emergency department
EHR: Electronic health record
GP: general practitioner
IADL: Instrumental activities of daily living
MDT: Multidisciplinary team (including health and social care providers)
